# Integrin β3/Akt signaling contributes to platelet-induced hemangioendothelioma growth

**DOI:** 10.1038/s41598-017-06927-0

**Published:** 2017-07-25

**Authors:** Rui Gu, Xin Sun, Yijie Chi, Qishuang Zhou, Hongkai Xiang, Dale B. Bosco, Xinhe Lai, Caixia Qin, Kwok-Fai So, Yi Ren, Xiao-Ming Chen

**Affiliations:** 1 Institute of Inflammation and Diseases, the First Affiliated Hospital of Wenzhou Medical University, Wenzhou, China; 2 Guangdong-Hong Kong-Macau Institute of CNS Regeneration (GHMICR), Joint International Research Laboratory of CNS Regeneration Ministry of Education, Guangdong Medical Key Laboratory of Brain Function and Diseases, Jinan University, Guangzhou, China; 3 Co-innovation Center of Neuroregeneration, Nantong, China; 4 Department of Biomedical Sciences, Florida State University College of Medicine, Tallahassee, FL USA

**Keywords:** Paediatric cancer, Cell growth

## Abstract

Hemangioendothelioma (HE) is a type of angiomatous lesions that features endothelial cell proliferation. Understanding the mechanisms orchestrating HE angiogenesis can provide therapeutic insights. It has been shown that platelets can support normal and malignant endothelial cells during angiogenesis. Using the mouse endothelial-derived EOMA cell line as a model of HE, we explored the regulatory effect of platelets. We found that platelets stimulated EOMA proliferation but did not mitigate apoptosis. Furthermore, direct platelet-EOMA cell contact was required and the proliferation was mediated via integrin β3/Akt signaling in EOMA cells. SiRNA knockdown of integrin β3 and inhibition of Akt activity significantly abolished platelet-induced EOMA cell proliferation *in vitro* and tumor development *in vivo*. These results provide a new mechanism by which platelets support HE progression and suggest integrin β3 as a potential target to treat HE.

## Introduction

Vascular neoplasms are tumors arising from blood vessel endothelial cells. Hemangioendothelioma (HE) defines vascular neoplasms that are characteristically between benign hemangiomas and malignant angiosarcomas^1^. HE comprises several clinical manifestations and histological hallmarks including papillary intralymphatic angioendothelioma, retiform HE, kaposiform HE, epithelioid HE, pseudomyogenic HE, and composite HE^2^. One specific form, Kaposiform HE, is frequently associated with Kasabach–Merritt syndrome (KMS), which features coagulopathy due to thrombocytopenia in infants^3^. To investigate the therapeutic targets of HE, the EOMA endothelial cell line, derived from a mixed HE arising in an adult mouse, was developed as a model of HE^4^. The EOMA cell line closely mimics the HE condition, responding to angiogenic regulators^5, 6^, inducing vessel formation, and promoting development of KMS in mice^7^. Therefore, a number of studies have utilized this cell line in order to examine the therapeutic potential of a variety of factors^8–11^. Moreover, examination of endogenous factors expressed by EOMA cells that increase angiogenesis such as insulin-like growth factor 2, 3-phosphoinositide-dependent kinase 1, transcription factor Prox1, and monocyte chemoattractant protein-1, has also provided important clues to HE progression^12–16^. However, to date, there is limited information on the *in vivo* microenvironment of HE and how it can modulate HE progression. Consequently, we sought to determine how microenvironmental factors influence HE development.

Platelets are one of the principal blood-borne contributors of angiogenesis. They are anucleate fragments of megakaryocyte cytoplasm which play essential roles in homeostasis and thrombosis under physiological and pathophysiological conditions^17, 18^. Recently, a great deal of information has been determined regarding the mechanisms underlying platelet-induced angiogenesis. Activated platelets released several trophic factors from specialized intracellular granules, such as vascular endothelial growth factor (VEGF), basic fibroblast growth factor (bFGF) and platelet-derived endothelial cell growth factor (PDGF), to support the survival and growth of endothelial cells^19–21^. Tumor cells can induce the activation of platelets, resulting in the promotion of tumor angiogenesis and the facilitation of cancer progression^22, 23^. Additionally, integrin β3, an abundant glycoprotein on the platelet plasma membrane, plays an important role in hypoxia-induced retinal angiogenesis and fetal angiogenesis, suggesting direct platelet-endothelium contact can mediate endothelial cell proliferation^24, 25^. Of note, integrin β3 is also highly expressed on endothelial cells and tumor cells contributing to several important cellular functions, for instance, migration, adhesion, angiogenesis and tumor growth^26, 27^. Alternatively, the internalization of platelets by endothelial cells may serve as another source of pro-angiogenic and anti-apoptotic effects^28^.

In the present study we utilized the EOMA cell line, a well-recognized cell model of HE, to investigate the influence of platelets on HE development. The proliferation and apoptosis of EOMA cells upon platelet treatment were examined. Furthermore, several of the aforementioned mechanisms driving platelet-induced angiogenesis were explored. This study illustrates the importance of platelets upon HE progression and suggests potential avenues for the therapeutic treatment of HE development.

## Results

### Platelets enhanced EOMA cell survival

**Figure 1:**
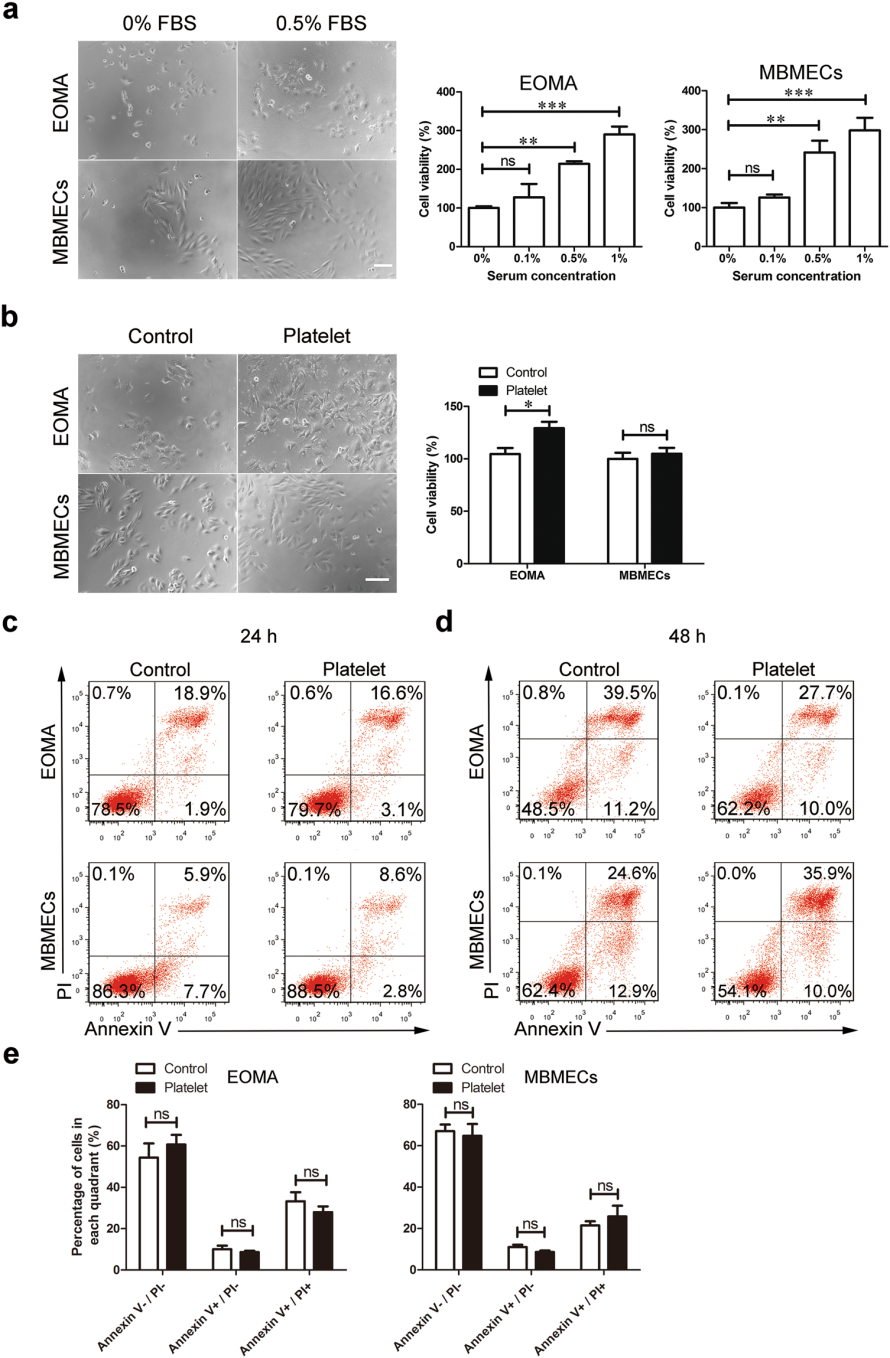
Platelet treatment increased the survival of EOMA cells without affecting cell apoptosis. (**a**) Effect of serum concentrations on the survival of EOMA cells and MBMECs. EOMA cells and MBMECs were cultured in medium with indicated concentrations of FBS for 72 hours. The cell viability was then assessed using the CCK8 assay. Representative images show the morphology of EOMA cells and MBMECs cultured with 0 and 0.5% serum for 72 hours. Scale bar, 50 μm. n = 5, one-way ANOVA. (**b**) Representative images and the cell viability of EOMA cell and MBMECs after platelet treatment for 72 hours. Scale bar, 75 μm. (**c**,**d**) Both EOMA cells and MBMECs were treated with platelets for (**c**) 24 hours and (**d**) 48 hours, stained with Annexin V/PI, and then evaluated via flow cytometry. (**e**) The 48-hour treatment of platelets did not affect apoptotic proportions of EOMA cells. n = 3, t-test. *P < 0.05; **P < 0.01; ***P < 0.001; ns, not significant.

To investigate their effect on HE, platelets were isolated from mouse blood and incubated with EOMA cells, a well-established cellular model of murine HE. We also employed mouse brain microvascular endothelial cells (MBMECs) from C57BL/6 J mice as a control to reveal tumor cell-specific activity in response to platelets. To exclude the influence of serum-derived factors, the viability of EOMA cells and MBMECs was examined using the Cell Counting Kit-8 (CCK8) assay with different FBS concentrations. We determined that 0.5% FBS supported modest and comparable growth in both EOMA cells and MBMECs (Fig. 1a). We therefore used this culture condition in subsequent studies. As shown in Fig. 1b, co-culture of EOMA cells with platelets for 72 hours significantly enhanced EOMA cell number approximately 125% of control, whereas MBMEC survival was not affected. This suggests that platelets affected EOMA cells specifically.

### Platelets did not affect EOMA cell apoptosis

We next wanted to determine if platelets increased cell number by inhibiting apoptosis. Using the well-established Annexin V/PI assay, we evaluated the apoptosis of EOMA cells and MBMECs co-cultured with platelets. After treatment with platelets for 24 or 48 hours, apoptosis was examined using flow cytometry (Fig. 1c,d). We determined that there was no significant change in either cell type of living, early apoptotic, and late apoptotic cell populations in response to platelets (Fig. 1e), suggesting that platelets do not increase EOMA cell resistance to apoptosis.

### Platelets stimulated EOMA cell proliferation

**Figure 2:**
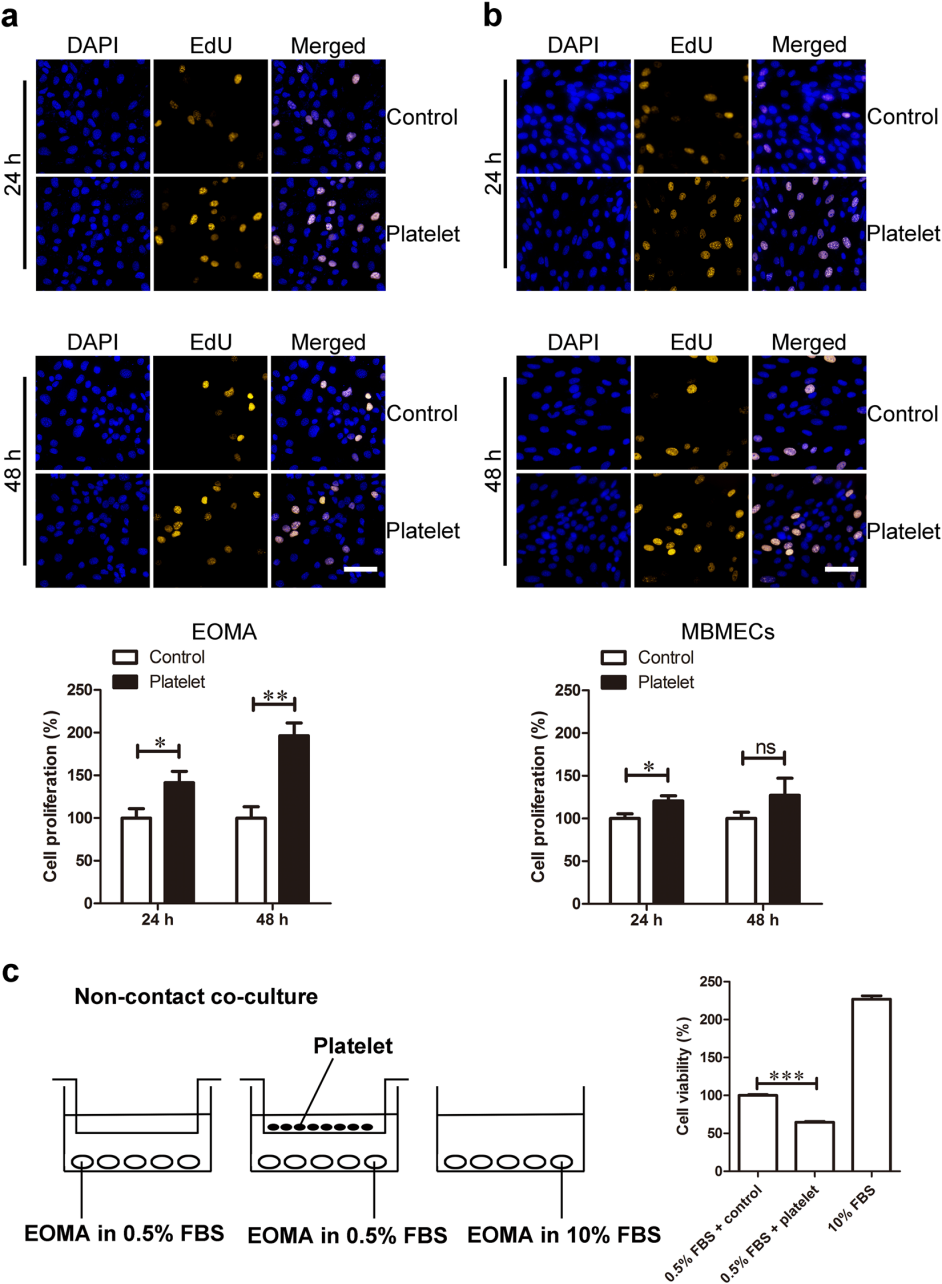
Platelet treatment induced EOMA cell proliferation. (**a**) EOMA cells and (**b**) MBMECs were incubated with platelets for either 24 or 48 hours. EdU (yellow) and DAPI (blue) staining was used to examine the cell proliferation. Scale bar, 60 μm. n = 5, t-test. (**c**) EOMA cells were incubated with platelets in a non-contact co-culture for 72 hours. The cell viability was examined by CCK8 assay. n = 4, one-way ANOVA. *P < 0.05; **P < 0.01; ***P < 0.001; ns, not significant.

Since apoptosis did not seem to be affected by platelet treatment, we asked if the apparent increase in cell survival reflects the up-regulation of proliferation. Thus, we performed 5-ethynyl-20-deoxyuridine (EdU) assays to quantify DNA synthesis, a hallmark of cell proliferation, in platelet treated EOMA cells. Treatment of platelets for both 24 and 48 hours significantly increased EdU incorporation into EOMA cell nuclei by approximately 150% and 200% of control, respectively (Fig. 2a). However, platelets did not induce significant EdU incorporation in MBMECs (Fig. 2b), which is in accordance with MBMEC survival results. We also examined if platelets could affect EOMA cells using non-contact co-culture. We found that platelets failed to increase EOMA cell viability in this situation (Fig. 2c). This result indicates that platelets may induce EOMAa cell proliferation in a cell contact-dependent manner. Consequently, our data demonstrates that platelets promote the proliferation of EOMA cells.

### EOMA cells did not induce pro-angiogenic activation of platelets

**Figure 3:**
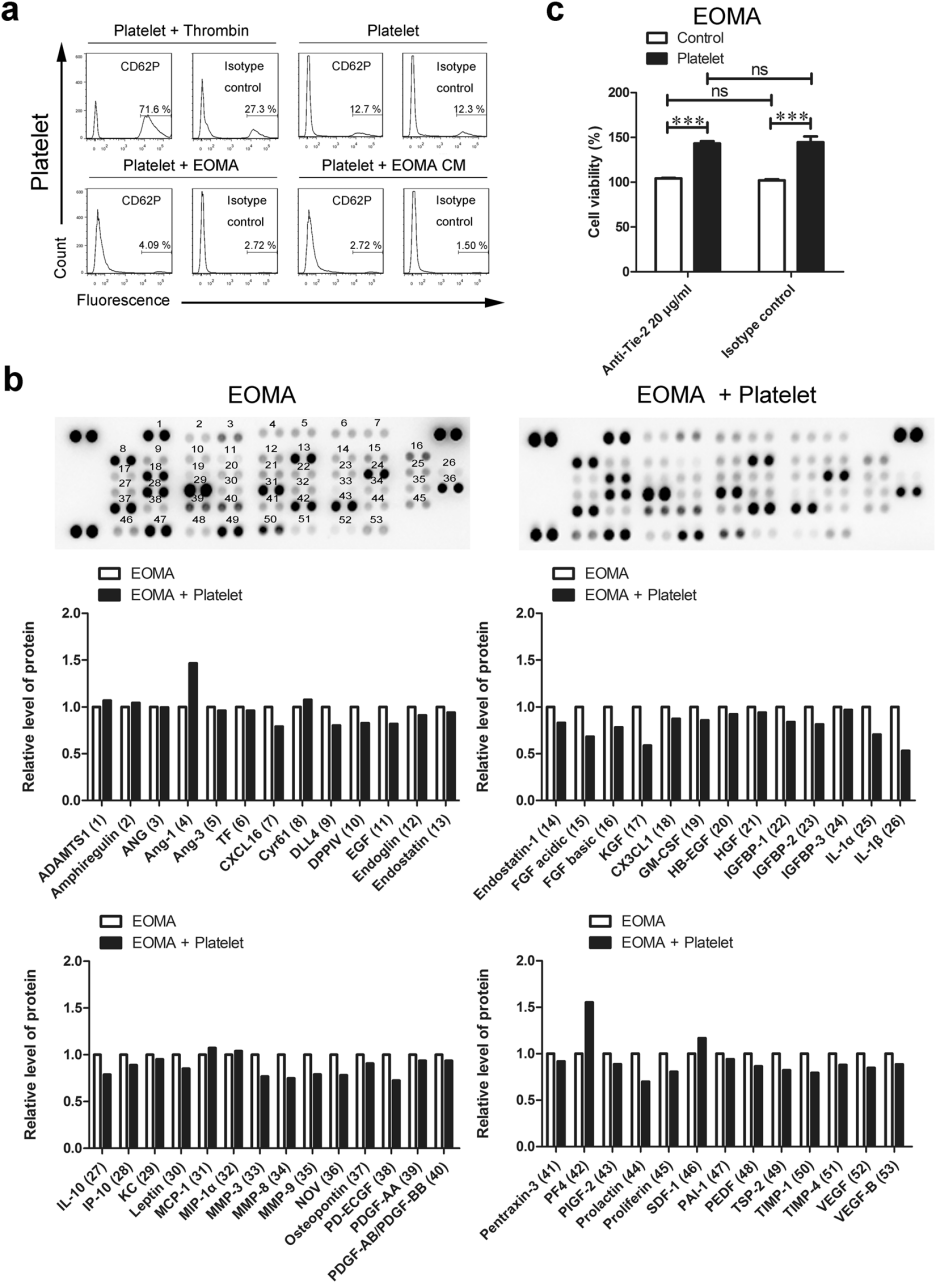
EOMA-platelet co-culture did not stimulate pro-angiogenic activation of platelets. (**a**) Platelets were co-cultured with either EOMA cells or MBMECs, or incubated in conditioned medium (CM) from either EOMA cells or MBMECs for 24 hours. Activation of platelets was then assessed by measuring the surface level of CD62P via flow cytometry. Thrombin was used as a positive control. (**b**) EOMA cells and MBMECs were cultured alone or with platelets for 24 hours. The levels of angiogenic factors in the culture medium were examined using the mouse angiogenesis antibody array. (**c**) EOMA cells were pre-treated with anti-Tie-2 antibody for 30 minutes and then incubated with platelets for another 72 hours. The cell viability was examined by CCK8 assay. n = 4, two-way ANOVA. ***P < 0.001; ns, not significant.

There is accumulating evidence indicating that platelets are activated to release pro-angiogenic factors in response to neighbor cells, including tumor cells^23, 29–31^. This may account for the observed platelet-induced increases in EOMA cell proliferation. To investigate this possibility, we analyzed the surface expression of CD62P (P-selectin), a marker of platelet activation, on platelets co-cultured with EOMA cells or incubated in EOMA conditioned medium (CM). As depicted in Fig. 3a, the surface level of CD62P, which was negligible on untreated platelets, was readily increased by thrombin, a commonly used platelet activator. However, no significant elevation of surface CD62P level was found on platelets in response to 24-hour platelet-EOMA co-culture or EOMA CM treatment (Fig. 3a). This suggests that EOMA cells may not be able to activate platelets. Next we investigated the release of angiogenic factors from platelets in response to EOMA cells. Except for pro-angiogenic factor angiopoietin-1 (Ang-1) and anti-angiogenic factor platelet factor 4 (PF4), we observed no obvious increase in angiogenic factor release (Fig. 3b). Of the two identified factors, Ang-1, and its receptor Tie-2, have been strongly linked to the process of angiogenesis^32^. Consequently, we further examined if Ang-1 had a role in EOMA cell promotion. We blocked the Tie-2 receptor activity on EOMA cells via neutralizing antibodies, and then treated these cells with platelets. However, blockage of the Tie-2 receptor failed to prevent platelet-induced EOMA cell survival (Fig. 3c). This suggests that EOMA cells do not stimulate pro-angiogenic activation of platelets and that Ang-1/Tie-2 signaling is not involved in platelet-induced EOMA growth.

### EOMA cells only had a limited capacity for platelet uptake

**Figure 4:**
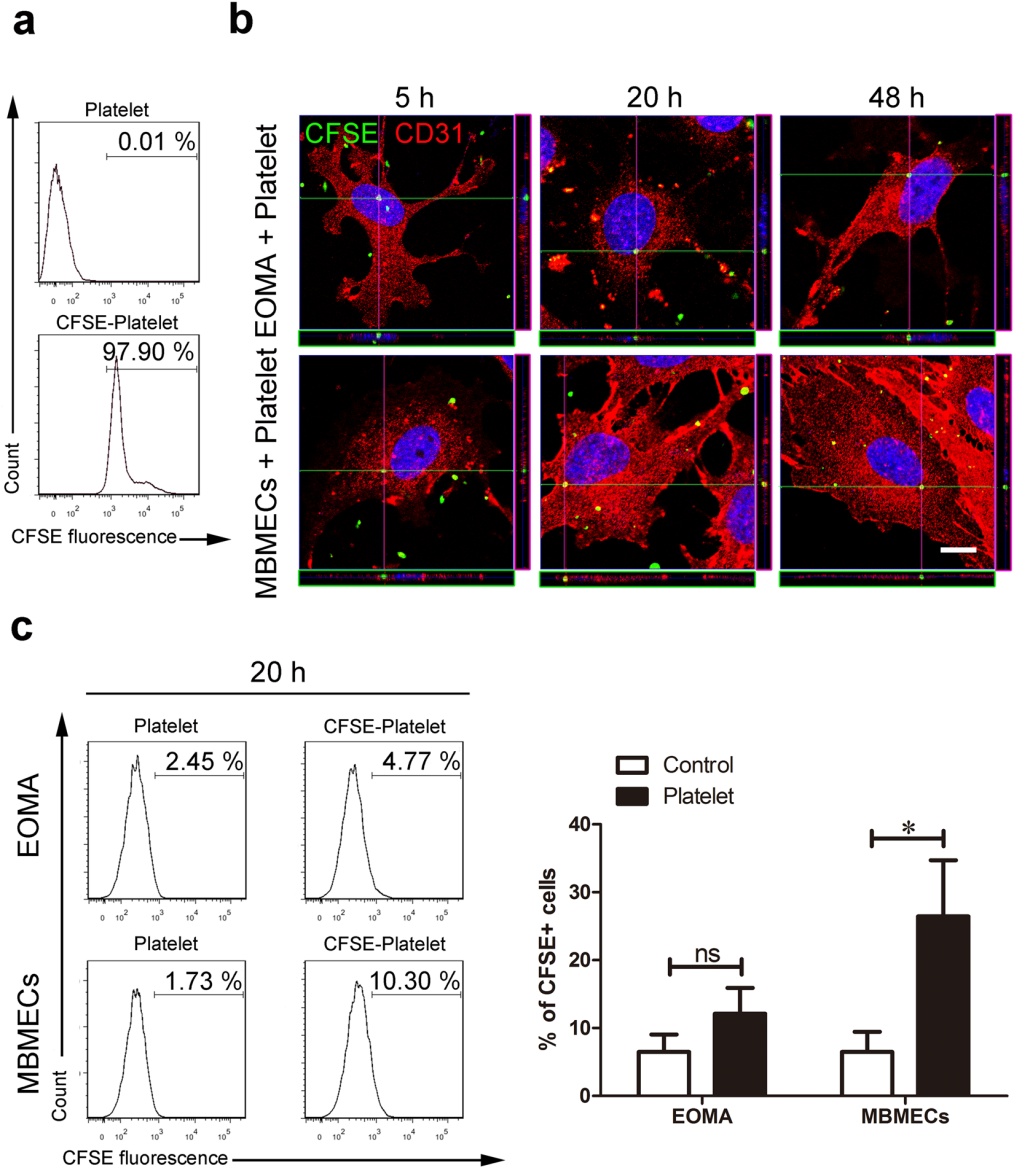
EOMA cells had a limited ability to internalize platelets. (**a**) Flow cytometric data showing the efficient CFSE labeling in platelets. (**b**) EOMA cells and MBMECs were incubated with CFSE^+^ platelets (green) for 5, 20, or 48 hours, and the engulfment of platelets by cells was detected by the localization of CFSE^+^ platelets within CD31^+^ cells (red). Scale bar, 10 μm. (**c**) EOMA cells and MBMECs were incubated with CFSE^+^ platelets for 20 hours and the amounts of internalized platelets were measured by flow cytometry. n = 3, t-test. *P < 0.05; ns, not significant.

It has been shown that endothelial cells are semi-professional phagocytes and possess the capacity to internalize platelets, stimulating angiogenesis^28^. However, the extent to which EOMA cells can internalize platelets and the subsequent effect on tumor angiogenesis are unclear. As such, we treated EOMA cells and MBMECs with carboxyfluorescein diacetate succinimidyl ester (CFSE)-labeled platelets (Fig. 4a) and evaluated the quantity of engulfed platelets. We found that EOMA cells, as well as normal MBMECs, were able to phagocytose platelets in a limited fashion (Fig. 4b). Nevertheless, flow cytometry analysis revealed that the increase of CFSE fluorescence in EOMA cells was undetectable after 20-hour platelet treatment (Fig. 4c), suggesting that EOMA cells do not robustly uptake platelets. Therefore, we speculate that the internalization of platelets is also not a driving force of EOMA cell proliferation.

### Integrin β3/Akt signaling was involved in platelet-induced EOMA cell proliferation

**Figure 5:**
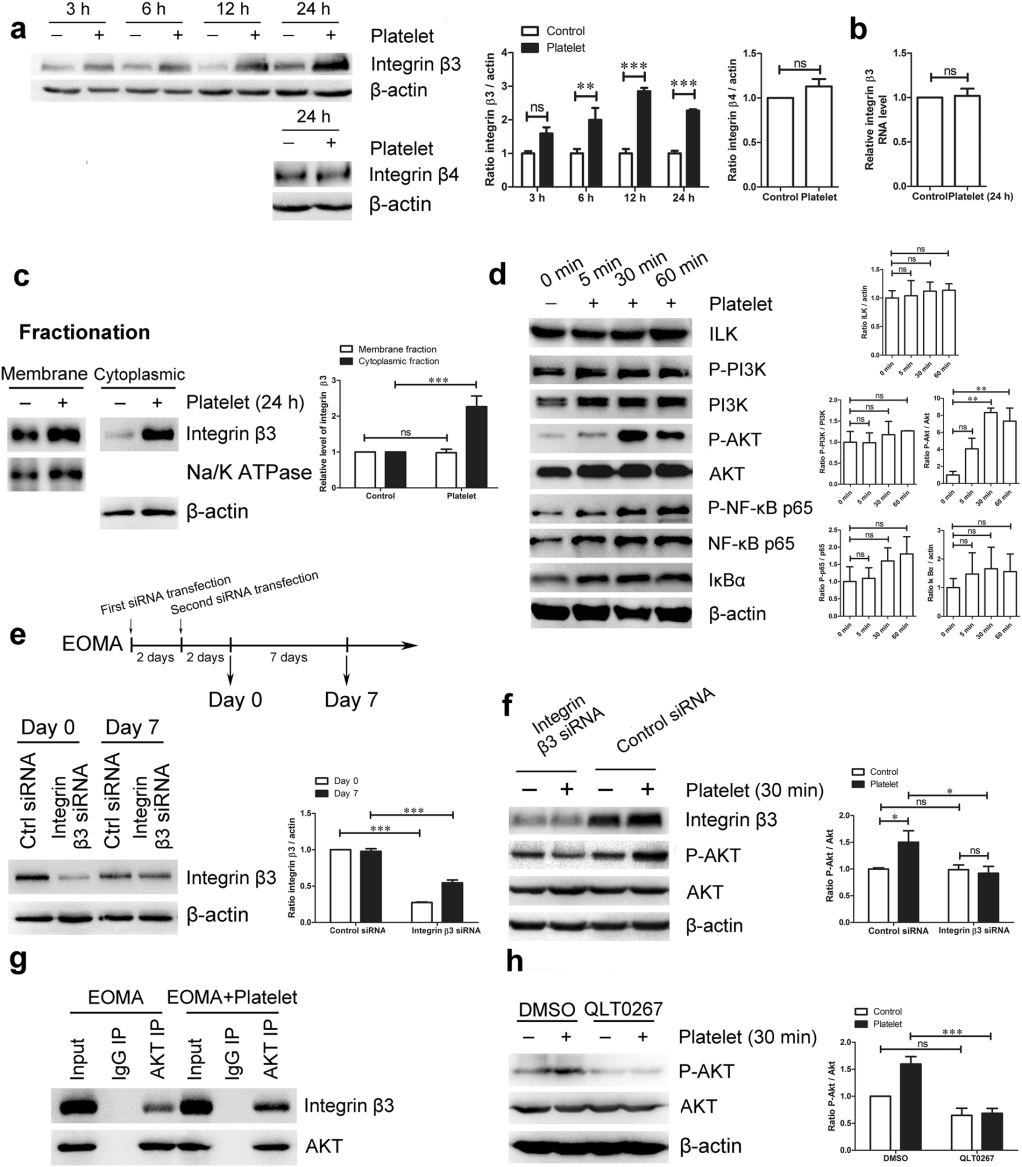
Integrin β3 was associated to platelet-induced Akt phosphorylation in EOMA cells. EOMA cells were treated with platelets for indicated times and (**a**) the total protein levels of integrin β3 and integrin β4, (**b**) RNA level of integrin β3, and (**c**) protein levels of integrin β3 in membrane and cytoplasmic fractions were examined by either Western blot or real-time PCR. Na/K ATPase and β-actin were used as loading controls. (**d**) EOMA cells were treated with platelets for specified times and the levels of ILK, phospho-PI3K, PI3K, phospho-Akt, Akt, phospho-p65, p65, and IκBα were assessed by Western blot. (**e**) EOMA cells were subject to siRNA transfections for 4 days and the level of integrin β3 at indicated times was examined by Western blot. (**f**) EOMA cells were transfected with control or integrin β3 siRNA for 4 days, and then treated with platelets for 30 minutes. The phosphorylation of Akt was assessed by Western blot. (**g**) EOMA cells were treated with or without platelets for 30 minutes then subject to immunoprecipitation using an Akt specific antibody. The interaction of integrin β3 with Akt was then assessed via Western blot. (**h**) EOMA cells were pre-treated with 10 μM QLT0267, and then incubated with platelets for 30 minutes. The phosphorylation of Akt was assessed by Western blot. n = 3–6, t-test, one-way or two-way ANOVA. *P < 0.05; **P < 0.01; ***P < 0.001; ns, not significant.

Next, we tested whether platelets can influence HE progression via activation of surface receptors on EOMA cells. It has been reported that integrins, abundantly expressed on the surface of endothelial cells, could contribute to contact-associated cell growth^33^. Following platelet treatment, the total protein levels of integrin β3, but not integrin β4, were significantly increased in a time-dependent manner (Fig. 5a). Interestingly, the up-regulation of total integrin β3 protein was not associated with increased integrin β3 mRNA expression (Fig. 5b), nor was it related to changes in surface expression of integrin β3 (Fig. 5c). Further analysis revealed that integrin β3 levels were only increased in the cytoplasmic fraction of EOMA cells (Fig. 5c), indicating that platelet treatment affects post-transcriptional regulation of integrin β3^34^.

We also observed significant phosphorylation of Akt in EOMA cells following short-term treatment of platelets (Fig. 5d), supporting the idea of contact-mediated EOMA proliferation. The PI3K/Akt/NF-κB pathway is a well-defined avenue through which the proliferation of endothelial cells is regulated^35–37^. However, the ratio of phosphorylated PI3K and NF-κB, was not significantly altered after platelet treatment (Fig. 5d), indicating that other unknown factors are participating in platelet-elicited Akt activation. To explore any possible association between integrin β3 and observed Akt phosphorylation, we performed siRNA-mediated knockdown of integrin β3 in EOMA cells, which effectively inhibited integrin β3 expression for up to 7 days (Fig. 5e). We observed that integrin β3 knockdown abolished Akt phosphorylation after platelet treatment (Fig. 5f). Immunoprecipitation assays also demonstrated that direct interaction between integrin β3 and Akt in platelet treated and untreated EOMA cells (Fig. 5g). To further investigate this connection we examined integrin-linked kinase (ILK), an important effector downstream of integrins. This kinase plays indispensable roles in multiple cellular functions including cell proliferation, migration, adhesions and signal transduction. Pre-treatment with the ILK-specific inhibitor, QLT0267, also prevented platelet-induced Akt phosphorylation (Fig. 5h). These results indicate the involvement of integrin β3/ILK signaling in platelet-associated Akt activation in EOMA cells.

**Figure 6:**
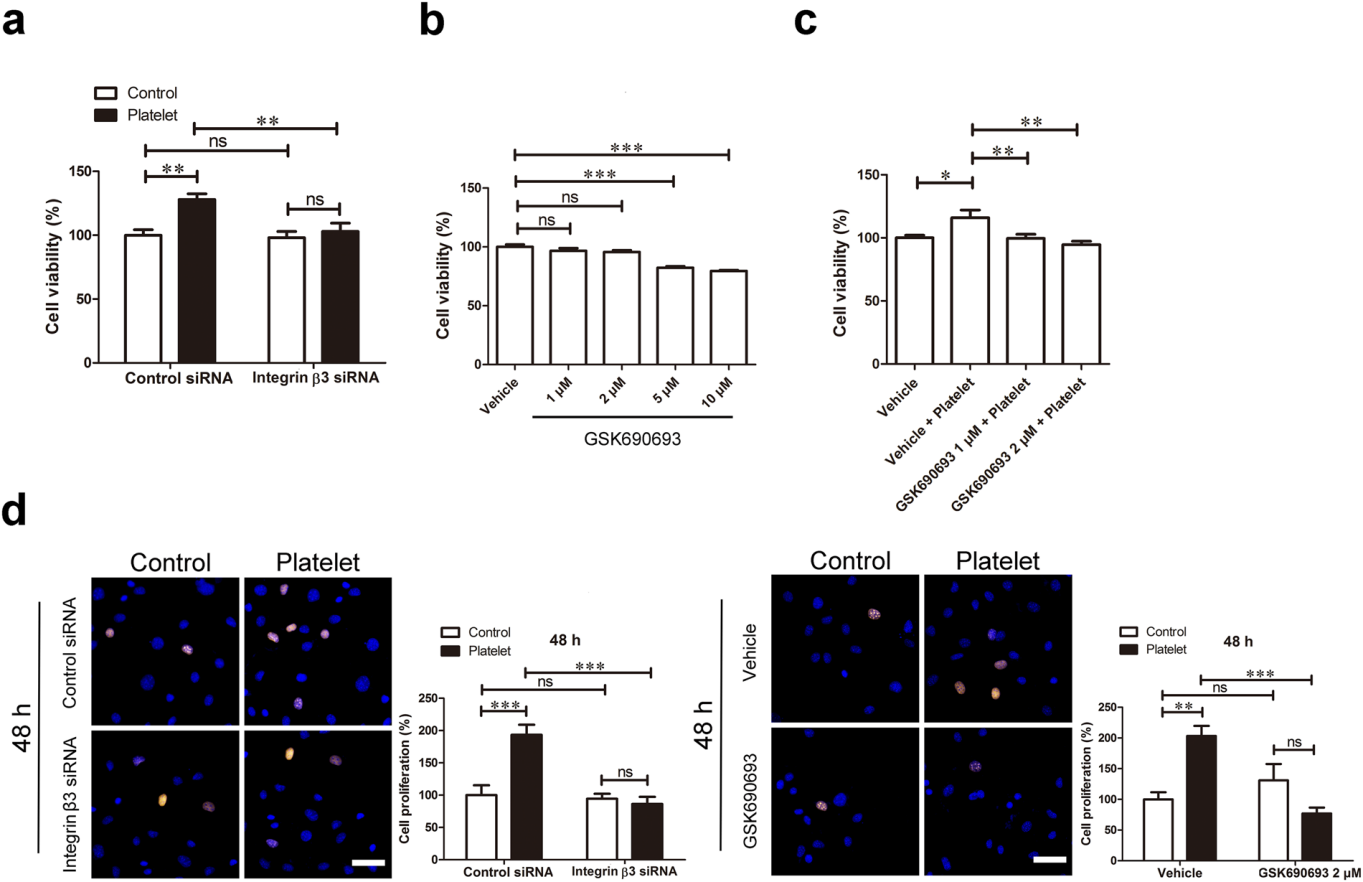
The integrin β3/Akt signaling contributed to platelet-induced EOMA cell proliferation. (**a**) EOMA cells were transfected with control or integrin β3 siRNA for 4 days, and then treated with platelets for another 72 hours. The cell viability was examined using the CCK8 assay. (**b**) The EOMA cells were incubated with indicated concentrations of Akt inhibitor GSK690693 for 72 hours. GSK690693 treatments with 1 and 2 μM did not significantly affect EOMA cell survival. (**c**) EOMA cells were pre-treated with Akt inhibitor GSK690693 for 3 hours, and then incubated with platelets for another 72 hours. The cell viability was examined using the CCK8 assay. (**d**) EOMA cells were either transfected with control or integrin β3 siRNA for 4 days, or pre-treated with GSK690693 for 3 hours, and then incubated with platelets for another 48 hours. The cell proliferation was assessed via the EdU assay. Scale bar, 60 μm. n = 3–5, one-way or two-way ANOVA. *P < 0.05; **P < 0.01; ***P < 0.001; ns, not significant.

Subsequently, we found that knockdown of integrin β3 inhibited platelet-induced EOMA cell proliferation (Fig. 6a). Using GSK690693, a potent Akt inhibitor which did not affect EOMA cell viability (Fig. 6b), we were able to suppress platelet-induced increases in EOMA cell proliferation (Fig. 6c). EdU assays confirmed that both the knockdown of integrin β3 and pharmaceutical inhibition of Akt activity abolished platelet-elicited EOMA cell proliferation (Fig. 6d). Taken together our results strongly suggest that direct platelet-EOMA cell contact and the activation of integrin β3/Akt signaling mediates platelet-associated proliferation of EOMA cells.

### Inhibition of integrin β3 expression and Akt activation attenuated HE growth *in vivo*

**Figure 7:**
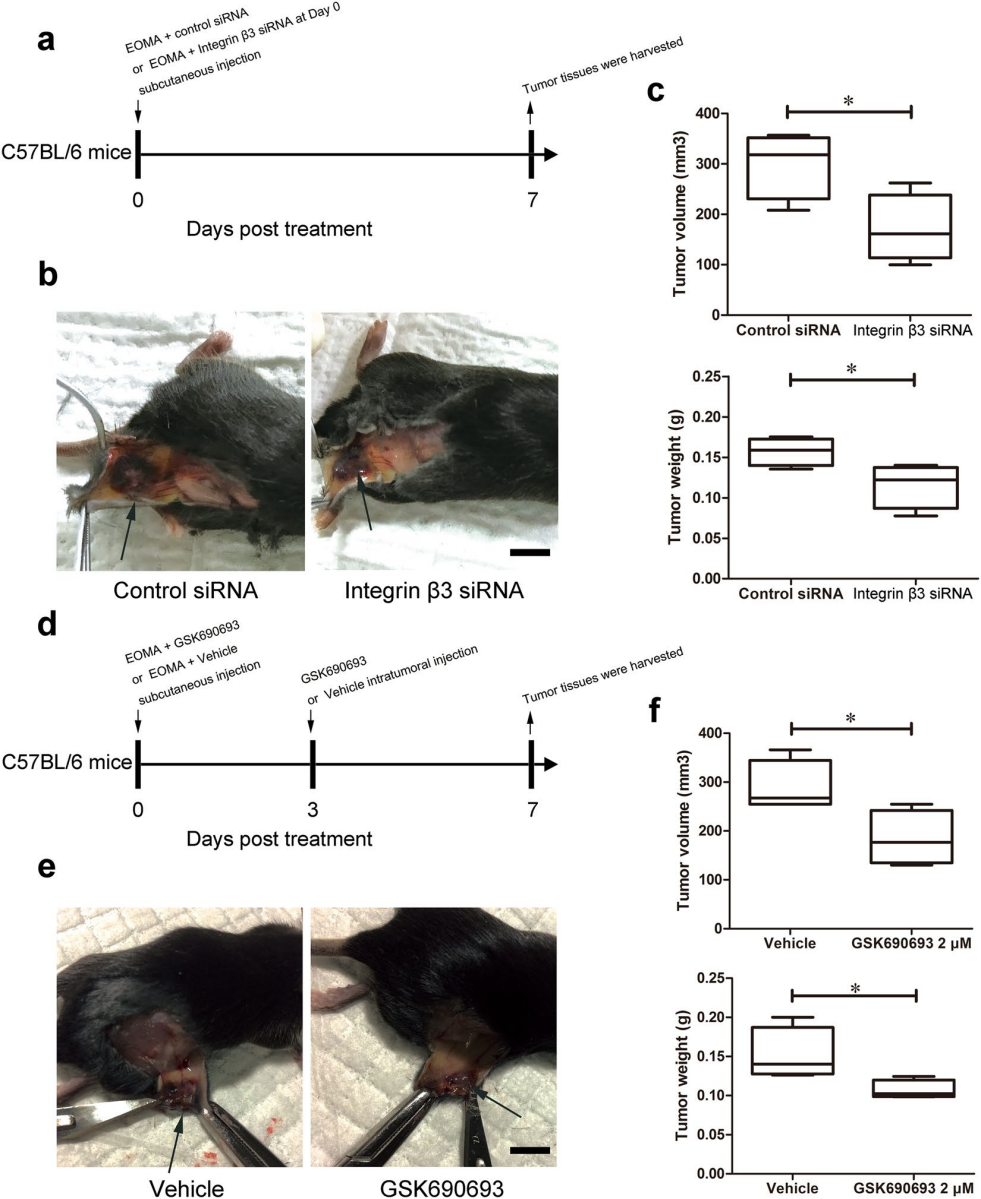
Knockdown of integrin β3 expression and inhibition of Akt activation attenuated HE growth *in vivo*. (**a**) EOMA cells were first transfected with control or integrin β3 siRNA for 4 days, then injected into C57BL/6 J mice on day 0. The tumors were collected on day 7. (**b**) Representative images showing subcutaneous growth of HE in response to the knockdown of integrin β3. Scale bar, 1 cm. Arrows indicated subcutaneous HE. (**c**) The tumor volumes and weights in response to the knockdown of integrin β3. (**d**) C57BL/6 J mice were injected with EOMA cells in the presence of GSK690693 or vehicle on day 0, then subjected to an additional intratumoral injection of GSK690693 or vehicle on day 3. Animals were sacrificed and the tumor tissues were harvested on day 7. (**e**) Representative images showing subcutaneous HE in response to Akt inhibition. Scale bar, 1 cm. Arrows indicated subcutaneous HE. (**f**) The tumor volumes and weights in response to Akt inhibition. n = 4, t-test. *P < 0.05.

Finally we determined if our *in vitro* results could be replicated *in vivo*. EOMA cells pre-treated with integrin β3 siRNA were administered to C57BL/6 J mice via subcutaneous injection and evaluated for HE development 7 days post-injection (Fig. 7a). Compared to the control, EOMA cells whose integrin β3 expression was inhibited developed smaller tumor masses, identified by reduced tumor volumes and tumor weights (Fig. 7b,c). Furthermore, subcutaneous injection of EOMA cells with GSK690693 (Fig. 7d) also resulted in smaller HE masses compared to the vehicle control (Fig. 7e,f). This suggests that both integrin β3 expression and Akt activity are critical determinants in platelet-regulated HE progression and are potential therapeutic targets.

## Discussion

While some investigations have shown that platelets elicited pro-apoptotic effects upon endothelial cells under specific pathological conditions^38, 39^, others showed that platelets could trigger multiple anti-apoptotic mechanisms, including the activation of Akt. Furthermore, literature shows that platelets can stimulate angiogenesis through various proliferative pathways such as VEGF, PDGF and bFGF signalings^40, 41^. In terms of tumorigenicity, platelets can promote the growth of various tumors, including bone, colorectal, and ovarian cancers^22, 42–44^. In the present study we observed that platelets have a proliferative, rather than pro-apoptotic impact on EOMA cells. However the effect of platelets may be influenced by numerous mediating mechanisms including, platelet-derived adhesion molecules (*e.g*., CD62P), platelet-released angiogenic factors (*e.g*., VEGF, PDGF), tumor cell membrane receptors (*e.g*., VEGF receptor, PDGF receptor, transforming growth factor-β receptor), and intracellular signaling molecules (*e.g*., Akt)^22, 42–44^. For example, platelets were found to inhibit the growth of murine lymphoma and prostate cancer cells^45^. Thus, the responses of tumor cells to platelets may cancer type specific.

Several studies have shown that tumors can activate platelets via promotion of aggregation and thrombosis^46–49^. Upon activation, platelets undergo de-granulation, releasing vast amounts of protein modulating important biological functions such as, thrombosis, angiogenesis, and would healing^29–31^. Wu *et al*. reported that lung cancer cells could induce platelets to release several pro-angiogenic factors, including IL-1α, GM-CSF, MMP-1 and VEGF, resulting in enhanced endothelial cell migration and capillary tube formation^23^. However, when we evaluated CD62P surface levels we did not observe significant activation by EOMA cells. Similarly, antibody array analysis revealed almost no up-regulation of pro-angiogenic protein release from platelets following tumor cell conditioning. Our data suggest that platelet activation and subsequent release of pro-angiogenic factors do not contribute to EOMA cell proliferation. We speculate that HE may have an inferior ability to activate platelets when compared to other types of tumors cells.

Endothelial cells possess a strong phagocytotic capacity for a variety of particulates, including aged and apoptotic cells^50^. Platelets and platelet-derived particles can also be effectively uptaken by endothelial cells^51, 52^, prolonging the survival of endothelial cells^28^. While we observed mild uptake by MBMECs, we observed for the first time that platelets are poorly internalized by EOMA cells, suggesting that tumor-derived endothelial cells exhibit impaired phagocytosis of platelets. This could be due to reductions in receptor-mediated recognition of platelets, or disrupted formation of phagocytotic vesicles. Further investigation is needed to determine why EOMA cells have a reduced capacity for platelet internalization.

Integrins are highly important to cell-cell contact^53, 54^, which can contribute to platelet-induced endothelial and tumor cell growth^55, 56^. Integrin β3 is abundantly expressed on endothelial cells and exerts essential effects on endothelial migration, adhesion, angiogenesis^27^, and importantly tumor growth^26^. For example, Integrin β3 plays a supportive role in melanoma survival *in vivo* and is a critical therapeutic target for the treatment of human melanoma-bearing animals^26^. Blockage of integrin β3 signaling can result in impaired angiogenesis and is anti-tumorigenic^24, 57^. The application of anti-integrin β3 antisera also helps ameliorate fetal and neonatal alloimmune thrombocytopenia-associated intracranial hemorrhage^24^. In line with the previously mentioned studies, we found that integrin β3 was involved in platelet-induced Akt phosphorylation and EOMA cell proliferation. However, the means by which integrin β3 activity on EOMA cells was regulated by platelets is still unclear. Platelets caused no change of the membrane proportion of integrin β3, suggesting the membrane trafficking of integrinβ3 was not influenced by platelets. Since platelets did not affect the transcription of *Itgb3* gene, the increased distribution of cytoplasmic integrin β3 by platelets could result from up-regulated mRNA translation or reduced protein degradation. More research is needed to illustrate such post-transcriptional regulations.

While platelet-induced EOMA cell proliferation was integrin β3- and Akt phosphorylation-dependent, inactivation of integrin β3 or Akt itself did not affect cell survival *in vitro*. The reason could be explained by the *in vitro* culture condition we applied, in which low concentration of serum may minimize basal cell growth along with minimal integrin β3 and Akt activation. When it comes to the *in vivo* situation, Akt is commonly hyperactivated in tumor cells, thus inactivation of Akt is of interest as a cancer treatment strategy^58^. Since the *in vivo* anti-tumor efficacy of Akt inhibitor GSK690693 has been shown in mice bearing breast tumor, lymphoma, endometrial tumor, ovarian carcinoma, and osteosarcoma^59, 60^, we also examined the role of GSK690693 in the treatment of HE in animals. As anticipated, GSK690693 significantly suppressed HE development *in vivo*, which supports a functional link between integrin β3, Akt activation, and HE progression. In summary, we demonstrate that platelets induce EOMA cell proliferation via cell-cell contact-based activation of integrin β3/Akt signaling. Furthermore, employing an *in viv*o HE model, our data indicate that the reductions of integrin β3 level and Akt activity mitigate HE progression, offering novel avenues for HE treatment.

## Materials and Methods

### Animals, cell lines, and reagents

Female C57BL/6 J mice (7–8 weeks of age) were purchased from the Jinan University Laboratory Animal Center. All outlined *in vivo* procedures were approved by the Institutional Animal Care and Use Committee of Jinan University. EOMA cells (CRL-2586), were obtained from the American Type Culture Collection (ATCC, Manassas, VA) and maintained in full medium (DMEM supplemented with 10% FBS and antibiotics) (Life Technologies, Grand Island, NY) at 37 °C, 5% CO_2_. MBMECs were prepared as previously described^61^ and cultured in full medium. All reagents were obtained from Sigma-Aldrich (St Louis, MO) unless otherwise indicated. QLT0267 was purchased from QLT, Inc. (Vancouver, Canada). The primary antibodies used were: rabbit-anti-IκBα (sc-371) from Santa Cruz Biotechnology (Dallas, TX); FITC-conjugated rat-anti-CD62P (#561923) and FITC-conjugated rat IgG1 isotype control (#553995) from BD Biosciences (San Jose, CA); rabbit-anti-integrin β3 (#13166), rabbit-anti-integrin β4 (#14803), rabbit-anti-β-actin (#12620), rabbit-anti-ILK (#3856), rabbit-anti-PI3K p85 (#4257), rabbit-anti-phospho-PI3K p85 (#4228), rabbit-anti-NF-κB p65 (#8242), rabbit-anti-phospho-NF-κB p65 (#3033), rabbit-anti-Akt (#4691), rabbit-anti-phospho-Akt (#4060) and normal rabbit IgG (#2729) from Cell Signaling Technology (Danvers, MA); rat-anti-CD31 (ab7388) and rabbit-anti-Na/K ATPase (ab76020) from Abcam (Cambridge, MA); goat-anti-Tie-2 (AF762-SP) and goat IgG isotype control (AB-108-C) from R&D systems (Minneapolis, MN).

### Platelet isolation

Mice were anesthetized with 4% trichloroacetaldehyde hydrate and the blood was collected via the orbital sinus. Nine volumes of fresh blood were mixed with 1 volume of anticoagulant citrate dextrose solution, then further mixed 1:1 with Tyrode’s solution. The mixture was centrifuged twice at 150 × g for 8 minutes, then the platelet-rich plasma collected from the supernatant was passed over a Sepharose 2B gel filtration column. The plasma-free platelets were eluted in Hepes-buffered modified Tyrode’s (HBMT) buffer and centrifuged at 650 × g for 8 minutes. Resulting pellets were resuspended in DMEM containing 0.5% FBS. Platelets were used at a final concentration of 5 × 10^6^ per mL.

### Cell viability assay

Cells were seeded into 96-well plates at a density of 1000 cells per well in full medium for 24 hours. The culture medium was then replaced with DMEM containing 0.5% FBS for 72 hours in the presence of platelets. Additional wells did not receive platelet treatment and served as a control. The cell viability was determined using the CCK8 assay (Dojindo molecular Technologies, Rockville, MD) according to the manufacturer’s instructions.

### EdU proliferation assay

Cells were seeded onto cover slips in 24-well plates at a density of 5000 cells per well in full medium for 24 hours, then cultured in DMEM with 0.5% FBS in the presence or absence of platelets for specified times. Cells grown in DMEM supplemented with 5% FBS were used as a positive control. After treatment, cells were exposed to 50 μM EdU (Ribobio, Guangzhou, China) for 5 hours, followed by fixation with 4% paraformaldehyde (PFA). Cells were then washed with PBS, permeabilized with 0.5% Triton X-100 for 10 minutes, and incubated with 300 μL of Apollo reaction cocktail for 30 minutes. Nuclei with yellow fluorescence were EdU-positive indicating proliferating cells. Total nuclei counts were determined via 4′,6-diamidino-2-phenylindole (DAPI) staining with blue fluorescence. The proliferation was stated as the ratio of EdU/DAPI double positive nuclei divided by total DAPI-positive nuclei.

### Annexin V-propidium iodide (PI) apoptosis assay

Cells were plated in 60-mm dishes containing full medium for 24 hours, and then treated with platelets in DMEM supplemented with 0.5% FBS for another 24 or 48 hours. Cells undergoing apoptosis were detected using the Annexin V-FITC apoptosis kit (BioVision, Milpitas, CA) according to the manufacturer’s instructions and analyzed using the FACS-Aria cytometer (BD Biosciences).

### Non-contact co-culture

EOMA cells were plated in 24-well plates containing full medium for 24 hours, and then incubated in DMEM containing 0.5% FBS with the inserts of Transwell chamber (0.4 µm pore size, Sigma) for another 72 hours. Platelets were placed in 100 µL of DMEM containing 0.5% FBS into the inserts. Cells grown in DMEM supplemented with 10% FBS were used as a positive control. Following co-culture, the cell viability was determined using the CCK8 assay.

### Platelet internalization assay

Platelets were labeled with CFSE, a cell-permeable fluorescent dye for 30 minutes, and then co-cultured with either EOMA cells or MBMECs for specified times. Un-ingested platelets were washed away with PBS. Internalized platelets were assessed via the detection of intracellular CSFE fluorescence using immunofluorescent staining and flow cytometry.

### Immunofluorescent staining

Cells were washed with PBS, fixed with 4% PFA and permeabilized with PBS containing 0.3% BSA and 0.1% Tween 20. Cells were incubated with primary and corresponding secondary antibodies at 4 °C (overnight) and room temperature (90 minutes), respectively. Image acquisition was performed using the Zeiss LSM700 confocal scanning microscope and the ZEN software (Carl Zeiss, Goettingen, Germany).

### Platelet activation assay

The platelets were treated as indicated, and the surface expression of CD62P, a marker of platelet activation, was probed by the FITC-conjugated primary antibody and analyzed using the FACS-Aria cytometer. The corresponding isotype control antibody was used as a negative control.

### Murine angiogenesis protein analysis

Culture media from EOMA cells and MBMECs, alone or co-cultured with platelets for 24 hours, were collected and centrifuged. The supernatants were used to detect the levels of released angiogenic factors using the Proteome Profiler Mouse Angiogenesis Array Kit (ARY015, R&D Systems) according to the manufacturer’s instructions.

### Integrin β3 knockdown

Cells seeded into 6-well plate were transfected with 300 pmol of either control or integrin β3 siRNA (sc-35677, Santa Cruz Biotechnology) in the presence of lipofectamine 2000 (Life Technologies) for 48 hours. Cells were then subjected to an additional transfection with 100 pmol of siRNA for 48 hours in order to maximize the knockdown effect.

### Western blot analysis

Following treatment, cells were lysed with RIPA buffer supplemented with protease and phosphatase inhibitor, then subjected to SDS-PAGE. Total proteins were then transferred onto a PVDF membrane (Immobilon-P; Millipore, Billerica, MA), blocked with 5% non-fat milk in 0.1% PBST, probed with primary antibodies (4 °C, overnight), and incubated with corresponding secondary antibodies (room temperature, 1 hour). After the enhanced chemiluminescence (ECL) with Luminata Forte Western HRP substrate (Millipore), the protein bands were imaged using the ChemiDoc Touch Imaging System (Bio-Rad) and the band intensities were analyzed with the ImageJ Software (https://imagej.nih.gov/ij).

### Real-time PCR

Following 24-hour platelet treatment, total RNA was extracted from EOMA cells using the Trizol reagent (Invitrogen) according to the manufacturer’s instruction. Reverse-transcription was carried out using 1 µg of total RNA, and the levels of *ITGB3* and *ACTB* mRNA were examined using SYBR Green Master Mix kit (Invitrogen) according to the manufacturer’s protocol. Forward primer of *Actb*: 5′-GGCTGTATTCCCCTCCATCG-3′, reverse primer of *Actb*: 5′-CCAGTTGGTAACAATGCCATGT-3′, Forward primer of *Itgb3*: 5′-CCACACGAGGCGTGAACTC-3′, reverse primer of *Itgb3*: 5′-CTTCAGGTTACATCGGGGTGA-3′.

### Cell fractionation

Cells were plated in 6-well plates containing full medium for 24 hours, and then treated with platelets in DMEM supplemented with 0.5% FBS for another 24 hours. Cytoplasmic and membrane fractions were separated using a cell fractionation kit (Cell Signaling Technology). These fractions were then analyzed by Western blot analysis.

### Immunoprecipitation

Following a 30-minute treatment with or without platelets, EOMA cells were lysed and immunoprecipitation was performed using a primary antibody directed against Akt (Cell Signaling Technology, #4691). Then a Pierce Crosslink IP Kit (ThermoFisher Scientific, Waltham, MA) was used according to the manufacturer’s instructions. Normal rabbit IgG was used as a negative control. Eluted proteins were resolved by the SDS-PAGE.

### Murine tumorigenesis

To generate HE, the injection of EOMA cells in mice was performed as previously described^12^ with minor modifications. Briefly, EOMA cells were collected, resuspended in DMEM medium (4.5 × 10^6^ cells in 200 μL) and subjected to dorsally subcutaneous injection in shaved C57BL/6 J mice. For knockdown of integrin β3, EOMA cells were first transfected with integrin β3 for 96 hours prior to injection. For inhibition of Akt, EOMA cells were co-injected with 2 μM GSK690693, followed by an additional intratumoral injection of 2 μM GSK690693 on day 3, when the tumors were palpable. Seven days post injection, mice were sacrificed. Following skin detachment HE tissues were harvested, and the extra fluid was removed to assess the tumor weights. The tumor volumes were measured using the formula volume = 0.52ab², where a and b indicated the long and short diameters of the tumors, respectively^9^.

### Statistical analysis

Data were presented as mean ± SEM. At a minimum each experiment was repeated in triplicates (n = 3–6). Student t-test, one-way analysis of variance (ANOVA) followed by Dunnett’s post hoc test, and two-way ANOVA followed by Bonferroni’s post hoc test were performed depending on experiment. Statistical significance was set at P value < 0.05.

### Ethics statement

All experiments and methods were conducted strictly with relevant guidelines and regulations of Jinan University. All animal procedures were performed according to the China’s animal welfare legislation for the protection of animals used for scientific purposes and approved by the Committee on the Ethics of Animal Experiments of Jinan University for care and use of laboratory animals. All efforts were made to minimize the number of animals and decrease their suffering.
