# Author Correction: Integrin β3/Akt signaling contributes to platelet-induced hemangioendothelioma growth

**DOI:** 10.1038/s41598-021-99149-4

**Published:** 2021-09-29

**Authors:** Rui Gu, Xin Sun, Yijie Chi, Qishuang Zhou, Hongkai Xiang, Dale B. Bosco, Xinhe Lai, Caixia Qin, Kwok-Fai So, Yi Ren, Xiao-Ming Chen

**Affiliations:** 1 Institute of Inflammation and Diseases, the First Affiliated Hospital of Wenzhou Medical University, Wenzhou, China; 2 Guangdong-Hong Kong-Macau Institute of CNS Regeneration (GHMICR), Joint International Research Laboratory of CNS Regeneration Ministry of Education, Guangdong Medical Key Laboratory of Brain Function and Diseases, Jinan University, Guangzhou, China; 3 Co-innovation Center of Neuroregeneration, Nantong, China; 4 Department of Biomedical Sciences, Florida State University College of Medicine, Tallahassee, FL USA

Correction to: *Scientific Reports* 10.1038/s41598-017-06927-0, published online 25 July 2017

This Article contains an error in Figure 6, where the Control and Platelet images for Control siRNA and Integrin beta 3 siRNA groups were mistakenly taken from the same original image file in panel (d).

The correct Figure 6 and accompanying legend appear below.

**Figure 6:**
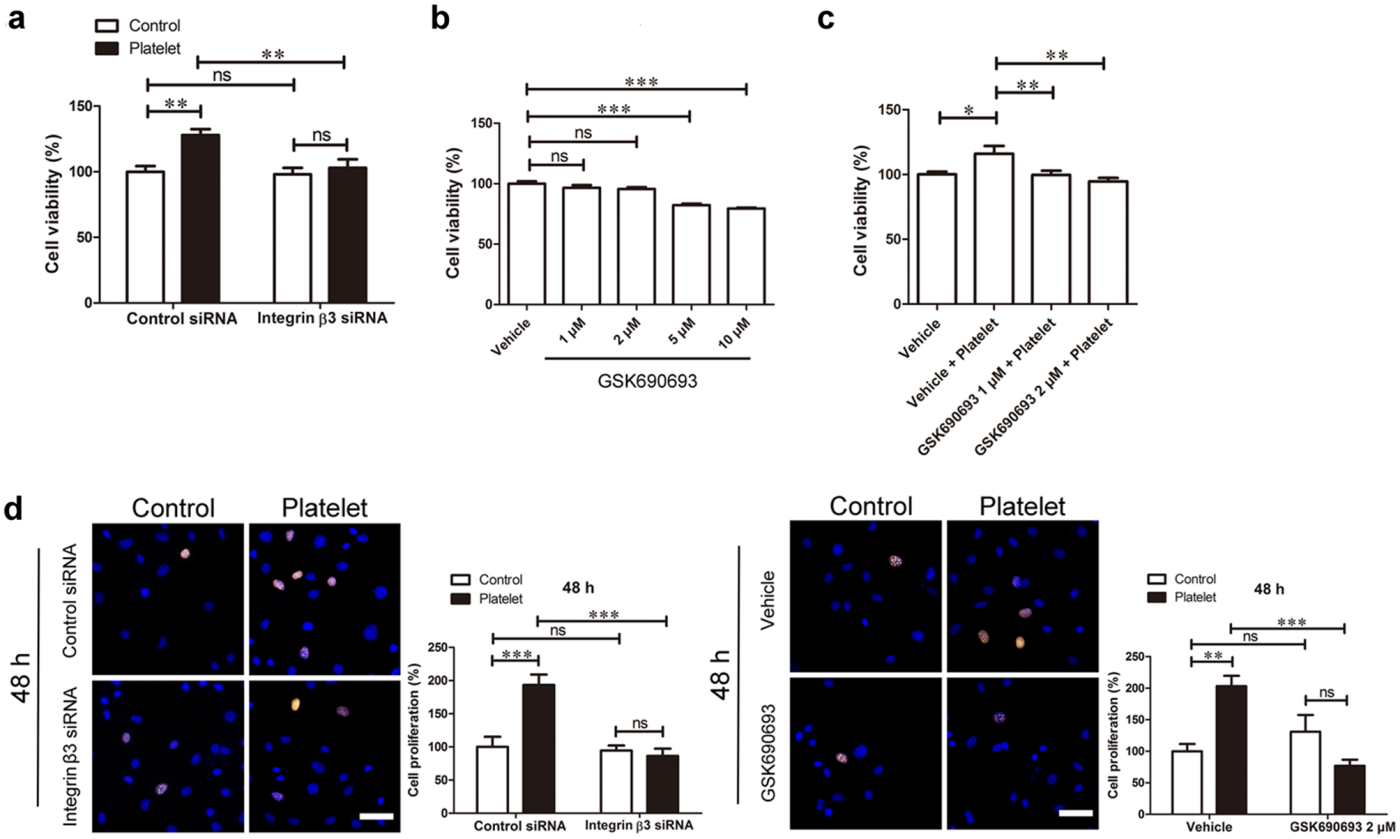
The integrin β3/Akt signaling contributed to platelet-induced EOMA cell proliferation. (**a**) EOMA cells were transfected with control or integrin β3 siRNA for 4 days, and then treated with platelets for another 72 hours. The cell viability was examined using the CCK8 assay. (**b**) The EOMA cells were incubated with indicated concentrations of Akt inhibitor GSK690693 for 72 hours. GSK690693 treatments with 1 and 2 μM did not significantly affect EOMA cell survival. (**c**) EOMA cells were pre-treated with Akt inhibitor GSK690693 for 3 hours, and then incubated with platelets for another 72 hours. The cell viability was examined using the CCK8 assay. (**d**) EOMA cells were either transfected with control or integrin β3 siRNA for 4 days, or pre-treated with GSK690693 for 3 hours, and then incubated with platelets for another 48 hours. The cell proliferation was assessed via the EdU assay. Scale bar, 60 μm. n = 3–5, one-way or two-way ANOVA. *P < 0.05; **P < 0.01; ***P < 0.001; ns, not significant.

Additionally, an incorrect email address for author Xiao-Ming Chen is quoted. Correspondence and requests for materials should be addressed to cxm@wmu.edu.cn.

